# Noise-Optimized Silicon Radiometers

**DOI:** 10.6028/jres.105.027

**Published:** 2000-04-01

**Authors:** George P. Eppeldauer

**Affiliations:** National Institute of Standards and Technology, Gaithersburg, MD 20899-8441

**Keywords:** chopped radiation, gain, noise, photocurrent, radiometer, sensitivity, silicon photodiodes

## Abstract

This paper describes a new, experimentally verified, noise analysis and the design considerations of the dynamic characteristics of silicon radiometers. Transimpedance gain, loop gain, and voltage gain were optimized versus frequency for photodiode current meters measuring ac and dc optical radiation. Silicon radiometers with improved dynamic characteristics were built and tested. The frequency-dependent photocurrent gains were measured. The noise floor was optimized in an ac measurement mode using photodiodes of different shunt resistance and operational amplifiers with low 1/*f* voltage and current noise. In the dark (without any signal), the noise floor of the optimized silicon radiometers was dominated by the Johnson noise of the source resistance. The Johnson noise was decreased and equalized to the amplified 1/*f* input noise at a 9 Hz chopping frequency and 30 s integration time constant, resulting in an equivalent root-mean-square (rms) photocurrent noise of 8 × 10^−17^ A. The lowest noise floor of 5 × 10^−17^ A, equal to a noise equivalent power (NEP) of 1.4 × 10^−16^ W at the 730 nm peak responsivity, was obtained at a 100 s integration time constant. The radiometers, optimized for ac measurements, were tested in a dc measurement mode as well. Performances in ac and dc measurement modes were compared. In the ac mode, a ten times shorter (40 s) overall measurement time was needed than in the dc mode (400 s) to obtain the same 10^−16^ A noise floor.

## 1. Introduction

Low-noise photocurrent measurements are needed for the detection of weak optical radiation [1]. Silicon photodiodes are well known for their excellent optical and electronic characteristics, and for their high sensitivity and stability [2, 3].

The noise and drift characteristics of silicon photodiode current meters were analyzed earlier at low (dc) frequencies [4]. High shunt resistance silicon photodiodes and operational amplifiers with large feedback resistors were used to achieve high photocurrent sensitivity. Operational amplifiers with very low input-bias current were used to minimize 1/*f* noise from the amplifier. The dominant Johnson noise was equalized to the 1/*f* amplifier noise by decreasing the electrical bandwidth to 1.25 mHz. The drift was also equalized to the noise floor by regulating the temperature of the Si radiometer within ± 0.02 °C of the operating 25 °C value [5]. The root-mean-square (rms) equivalent photocurrent of the measured noise floor was 10^−16^ A. The time of the measurement was 400 s. When the bandwidth was increased to 300 mHz, the noise increased and ranged between 0.6 fA and 1 fA. The factor of 10 noise increase was measured for the photodiode with the largest time constant (shunt resistance times junction capacitance). This output noise increase was due to a frequency-dependent noise-boosting effect in the voltage gain of the photocurrent meter.

The dynamic (ac) characteristics of photodiode current meters were analyzed in transimpedance amplifier applications [6,7,8]. The fundamental gain equations were summarized recently [9] to optimize signal (transimpedance) gain, voltage gain, and loop gain versus frequency. The gain equations can be used to optimize both low-frequency (dc) and modulated/chopped (ac) photocurrent meters. The feedback impedances of the selected operational amplifiers were matched to the impedance of the selected silicon photodiode in the analyzed ac photocurrent meters. The signal roll-off point of large-area silicon photodiode current meters could be increased to 80 Hz even at a signal gain of 10^9^ V/A. The suggested silicon radiometer converts the optical radiation signal (chopped with 8 Hz) into an ac voltage signal which has negligible amplitude distortion and signal phase shift. The optimization of the frequency-dependent (three) gains was extended to Ge, InGaAs [10], and InSb [11] photodiodes as well.

Optimization of the dynamic (frequency-dependent) characteristics of silicon radiometers will not necessarily optimize the sensitivity of the meters. The fundamental gains and the noise floor have to be optimized together to get the best performance. This paper shows the proper method of analysis for performing this task in the ac measurement mode. The design considerations for both the dynamic characteristics and the noise performances are experimentally verified. Also, the performances of ac and dc silicon radiometers are compared.

## 2. Gain Equations of Photodiode Current Meters

**Fig. 1:**
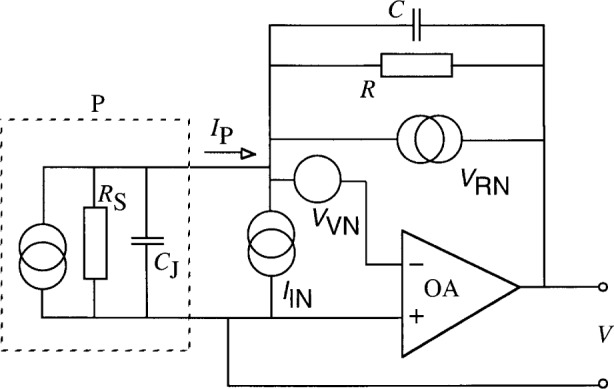
Equivalent circuit of photodiode radiometers. The dashed line represents photodiode P. *R* and *C* are the feedback components of the operational amplifier OA.

The equivalent circuit of photocurrent-to-voltage converters is shown in Fig. 1. The photocurrent *I*_P_ from photodiode P is converted into a voltage *V* at the output of the operational amplifier OA. *R*_S_ is the shunt resistance and *C*_J_ is the junction capacitance of P. *R* is the feedback resistance and *C* is the feedback capacitance of the OA. The photocurrent-to-voltage conversion can be described by the transimpedance gain [9]:

$$A_{I}=\frac{V}{I_{P}}=R\frac{1}{1+j\omega RC}.$$ (1)

Equation (1) shows that the dc signal gain (at *ω* = 0), which is the ratio of the output voltage *V* to the input photocurrent *I*_P_, is equal to *R*. The frequency-dependent signal response is determined by the integrating time constant *τ*_1_ = *RC* of the feedback impedance. Equation (1) also describes the gain for the input current noise *I*_IN_ of the circuit. Because Eq. (1) does not show the frequency dependent contribution of OA to the signal response, the equation works only if the roll-off time constant of the OA open-loop gain *τ*_i_ < *τ*_1_. Otherwise, the OA frequency-dependent response gives an unwanted limitation for the signal response.

Similarly to Eq. (1),

$$\frac{V}{I_{P}}=R\frac{1}{1+G^{-1}},$$ (2)

where *G* is the loop gain. The photocurrent-to-voltage conversion *R*, which is the signal gain, will be accurate only if *G* >> 1 at the signal frequency. This is a very important design requirement for the analog control loop.

The loop gain, which is the product of the OA open loop gain *A* and the feedback attenuation *β*, can be written as

$$G=A_{0}\beta_{0}\frac{1}{1+j\omega \;\tau_{i}}\frac{1+j\omega \;\tau_{1}}{1+j\omega \;\tau_{2}},$$ (3)

where *A*_0_ is the dc open loop gain and *β*_0_ = *R*_S_/(*R*_S_ + *R*). The quantity *τ*_1_ = *RC* was the integrating time constant in Eq. (1). Here, in Eq. (3), *τ*_1_ is a differentiating time constant. The integrating time constant *τ*_2_ here is the product of the parallel resistances and the parallel capacitances:

$$\tau_{2}=\frac{R\;R_{S}\;C+R\;R_{S}\;C_{J}}{R+R_{S}}=\frac{R\;R_{S}}{R+R_{S}}(C+C_{J}).$$ (4)

*τ*_i_ is always an additional integrating type time constant.

The closed loop voltage gain, which is the reciprocal of *β*[12], determines the amplification for the input voltage noise *V*_VN_:

$$A_{V}=A_{V_{0}}\frac{1+j\omega \tau_{2}}{1+j\omega \tau_{1}}$$ (5)

where

$$A_{V_{0}}=\frac{R_{S}+R}{R_{S}}$$ (6)

is the dc voltage gain.

In contrast to Eq. (3), *τ*_1_ is an integrating and *τ*_2_ is a differentiating time constant in Eq. (5). *V*_RN_ is the Johnson (resistor) noise. This “white” noise shows up directly at the OA output (without any amplification). The current noise *I*_IN_ is caused by the fluctuation of the OA input current. Both *I*_IN_ and *V*_VN_ have “white” and 1/*f* noise components superimposed on each other.

The photocurrent *I*_P_ produces a shot noise *I*_PN_ which is not shown in Fig. 1. The current *I*_PN_ is converted to the OA output similarly to *I*_P_. This current noise component is (2*eI*_P_Δ*f*)^1/2^, where *e* is the elementary electron charge, 1.60 × 10^−19^ C, *I*_P_ is the photocurrent, and Δ*f* is the electrical bandwidth. A 10^−14^A photocurrent produces an rms current noise of *I*_PN_ = 7.1 × 10^−18^ A at Δ*f* = 16 mHz, which corresponds to an integration time constant of 10 s.

At the OA output, the signal-produced voltage *V* has to always be much larger than the superimposed total noise voltage originating from the above four noise components.

## 3. Design of Dynamic Characteristics

A test silicon radiometer was built that satisfies the three gain requirements discussed above with high photocurrent and loop gains and low voltage-gain at the signal frequencies.

According to the suggestion in our previous work [9], the Hamamatsu Model S5226-8BQ^1^ 1/3 cm^2^ area photodiode was selected and tested with *R*_S_ = 2.2 GΩ and *C*_J_ = 410 pF. The large shunt resistance kept *A*_V_ low. The small junction capacitance tuned the *f*_2_ = 1/(2π*τ*_2_) roll-up point in the voltage-gain curve to high frequencies, resulting in decreased noise-boosting effect. The Burr-Brown OPA111BM operational amplifier was selected because of its high dc open loop gain (*A*_0_ = 10^6^) and low 1/*f* noise. The dynamic characteristics below were designed with typical manufacturer’s data regarding *C*_J_, *A*_0_, and the 3 dB roll-off frequency of the OA open-loop gain.

The largest feedback resistor used in these tests was *R* = 10^10^ Ω. In order to obtain a high enough signal roll-off frequency *f*_1_ = 1/(2πτ_1_) at this signal gain, a small stray capacitance of *C*_s_ = 1.25 pF was realized. The overall feedback capacitance was *C* = *C*_s_ + *C*_f_, where *C*_f_ was the externally connected feedback capacitor. *C*_f_ was selected for all gains (except *R* = 10^10^ Ω) to obtain a signal roll-off frequency of at least 10 times the chopping frequency.

**Fig. 2:**
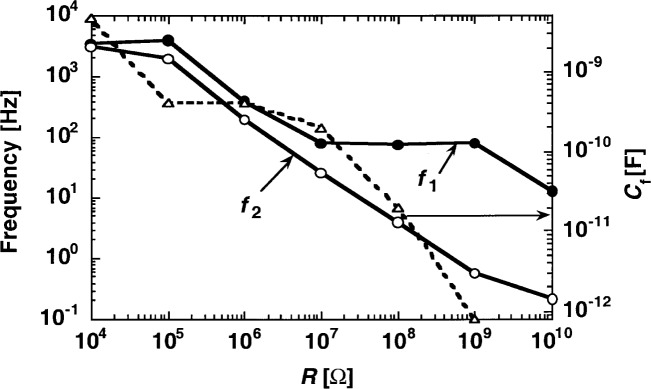
Time constants-produced frequency roll-off and roll-up points of the test radiometer calculated for the decadic signal gains. The external feedback capacitor versus feedback resistor is shown by the dashed curve. The points are connected to help visual separation of the curves.

Figure 2 shows *C*_f_, *f*_1_, and *f*_2_ versus *R* for the test radiometer. The *f*_1_ and *f*_2_ curves were calculated from *τ*_1_ and *τ*_2_ of Sec. 2. As is shown in Eq. (4)*f*_2_ depends on *C*. A change in *C*_f_ will modify the starting value of *f*_2_. *C*_f_ was selected for all signal gains to obtain a high enough *f*_1_ for chopping frequencies between 7 Hz and 10 Hz. The frequency *f*_1_ was decreased by increasing *C*_f_ in all signal gains (other than the highest) to tune *f*_1_ close to *f*_2_ as much as possible. This requirement is important for minimizing noise voltage amplification and maximizing loop gain for the signal frequencies. At the same time, *f*_1_ had to be high enough to keep the signal bandwidth of the radiometer broad enough for all signal-gain selections. This is an important requirement to avoid distortion of the signal shape and to make the signal-phase-shift negligibly small. At full-frequency compensation, where *τ*_1_ = *τ*_2_, the differentiating time constant cancels (compensates) the integrating time constant. This way, the loop gain can be increased and the voltage gain can be decreased for the signal (chopping) frequencies. For gain selections of *R* << *R*_S_ the 1 = 2 condition requires *C* >> *C*_J_, resulting in large *C*_f_ values. The capacitance *C*_f_ will not be too large if *C*_J_ is small. Full frequency compensation was made at *R* = 10^4^ Ω using *C*_f_ = 4.7 nF. No frequency compensation was made for *R* = 10^10^ Ω to keep *f*_1_ high. Full compensation for the high signal gains would make the radiometer very slow. Partial compensations satisfied the three gain requirements for all the other signal gains.

**Fig. 3:**
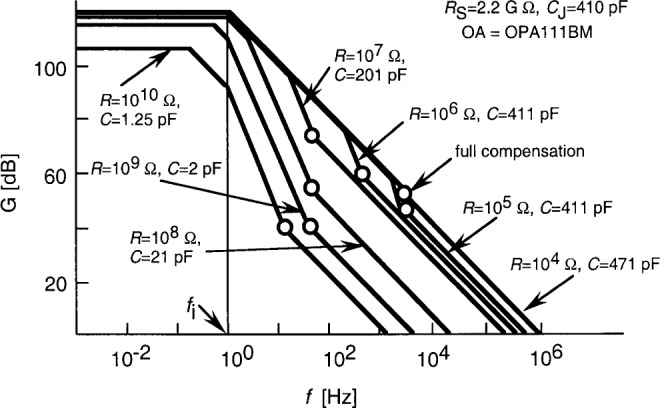
Loop-gain characteristics of the test silicon (S5226-8BQ) radiometer. The open circles show the signal 3 dB roll-off points as they are matched to the loop-gain characteristics.

Figure 3 shows the frequency-dependent loop-gain characteristics of the above test radiometer. This is a Bode plot [13] where the characteristics can be constructed quickly and fairly accurately by approximating the curves with piecewise linear regions. The value of *G* at low frequencies is equal to *A*_0_*β*_0_. The first two roll-offs are at *f*_2_ and *f*_i_; *f*_1_ determines the roll up. This is the frequency where the signal 3 dB roll-off points (shown with open circles) are attached to the loop-gain curves (solid lines). At *R* = 10^10^ Ω, the signal 3 dB point is 13 Hz without any frequency compensation (*C*_f_ = 0). At *R* = 10^9^ Ω, *C*_f_ = 0.8 pF will produce *C* = 2 pF with a roll-off frequency of *f*_1_ = 78 Hz. As a result of the high open-loop gain of the OPA111BM operational amplifier, the loop gains at these two highest signal gains are 100 (40 dB). Partial frequency compensations were enough for the other signal gains to obtain high enough loop gains at the signal 3 dB roll-off points. For these cases, *f*_1_ = 75 Hz, or higher, was selected. The full frequency compensation at the lowest signal gain was made as a design example.

**Fig. 4:**
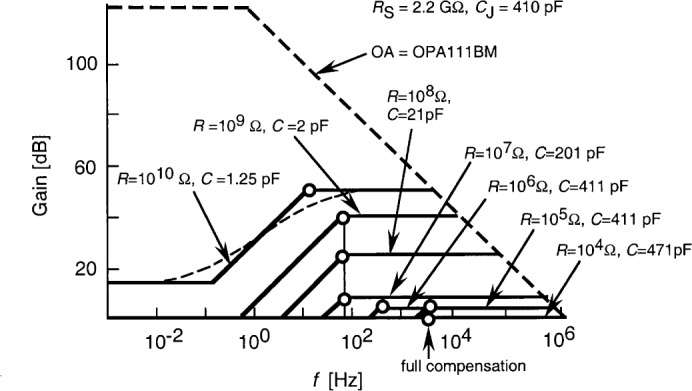
Closed-loop voltage-gain characteristics (solid lines) of the test silicon (S5226-8BQ) radiometer. The thick dashed lines show the open-loop gain of the OA. The open circles are the signal 3 dB roll-off points as they are matched to the voltage-gain characteristics.

Figure 4 shows the closed-loop voltage-gain characteristics (solid curves) of the test silicon radiometer. The open-loop gain curve of the OA is shown with thick dashed lines. The roll up here is determined by *f*_2_. The roll-off is produced by *f*_1_. At the highest gain, *f*_1_ is relatively far from *f*_2_, producing a gain increase of about two orders of magnitude. The real shape of this gain change is shown (for *R* = 10^10^ Ω only) with the thin dashed curve. The noise boosting at a chopping frequency of 9 Hz is still significant.

## 4. Measurement of Photocurrent Gains

**Fig. 5:**
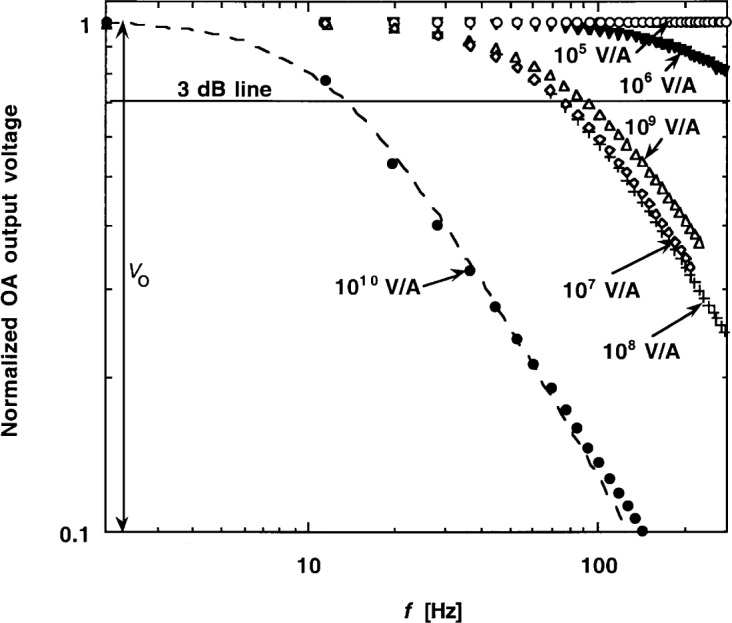
Measured frequency-dependent signal-gain curves of the test silicon radiometer for different signal-gain settings; *V*_0_ is the dc output voltage of the radiometer.

The frequency-dependent signal (transimpedance) gain of the test silicon radiometer was measured for all (except for the 10^4^ V/A) signal-gain selections. The stable broad-band radiation of a 100 W tungsten halogen lamp was measured using a chopper. The frequency of the chopper was tuned from about 10 Hz to 300 Hz by computer control. A chopper-synchronized, sine-wave measuring lock-in amplifier was connected to the output of the radiometer. The measured output voltage *V* curves are normalized and shown versus chopping frequency *f* in Fig. 5. At low (dc) frequencies, the output voltage *V*_0_ is not attenuated. The 3 dB line on the graph shows where the signal-gain curves reach the 70.8 % (3 dB attenuation) value of *V*_0_. The signal attenuation, according to Eq. (1), is caused by the *τ*_1_ = *RC* time constant, which is different for all signal gain selections. There is no monotonic progression in the measured curves because the 3 dB signal frequencies agree with the design values (*f*_1_) in Sec. 3. The 3 dB frequency for 10^9^ V/A gain was larger (about 90 Hz) than the designed 78 Hz because the very small (calculated) external feedback capacitor (*C*_f_ = 0.8 pF) was not connected. For the most sensitive signal gain the 3 dB point was calculated from a curve fit to the measured data points. The fit is shown with the dashed curve. The fit equation is:

V(f)=V01+(ff1)2. (7)

The 3 dB point from the fit was 12.9 Hz. The response at 2 Hz is a rough extrapolation to *V*_0_ measured with the other signal gain selections. For a chopping frequency of 9 Hz the amplitude attenuation at the 10^10^ V/A signal-gain selection will be about 17 %. Because the operating point is on the slope of the roll-off curve, the chopping frequency should be stabilized for this gain selection. The quantity *V*_0_ should be reported instead of *V* versus frequency if different chopping frequencies are used. The graph also shows that for the other gain selections the signal responses are flat around the 9 Hz chopping frequency.

## 5. AC Measurement of Dark Noise

In ac photocurrent measurements, amplifier drifts do not contribute to measurement uncertainties. It is the noise floor that determines the sensitivity limit. The dark noise gives basic information about the noise performance of a radiometer. In order to make high-sensitivity measurements, the dark noise has to be kept as low as possible. In our noise tests the output of the test radiometer was connected to the inputs of chopper-synchronized lock-in amplifiers. The photodiode was covered with a light-tight cap in order to measure the dark noise. The total output noise of the radiometer (dark) was measured at a chopping frequency of 9 Hz with an electrical bandwidth determined by the low-pass filter at the output of the lock-in amplifier. In these ac measurements the signal magnitudes *M* were calculated from the lock-in *X* and *Y* (filtered) output voltages using the mathematical vector sum process (*M* = (*X*^2^ + *Y*^2^)^1/2^). When measuring noise (or a small signal in the noise), this process rectifies the noise, causing residual output offset. In order to estimate the noise measurement uncertainty caused by this problem, several dark noise tests were repeated and evaluated in a different way, i.e., by calculating the noise fluctuations in both *X* and *Y*. The lock-in *X* and *Y* output noise voltages and the magnitudes are shown in Table 1 for 30 s and 1 s integration time constants. The *X* and *Y* pairs were always simultaneous measurements. The *M* values, at a given time constant, were measured consecutively to the measurements of *X* and *Y*. Each reported noise value was calculated as the standard deviation of at least 20 readings. The measurement results show that the noise of *M* can be 38 % lower than the noise of *X* or *Y*, which are free of the residual output offset. The uncertainty of our noise magnitude measurements is dominated by this 38 % (coverage factor *k* = 2) relative expanded uncertainty.

### 5.1 Noise Floor Optimization

The noise floor of the test silicon radiometer was optimized in the ac measurement mode using four photodiodes with different shunt resistances and three operational amplifiers with low 1/*f* voltage and current noise. The feedback capacitors were not changed for the different photodiode-amplifer combinations. Table 2 shows the model numbers and the impedances of the tested photodiodes. All tested photodiodes had an active area of 1/3 cm^2^. The models in Table 2 were chosen in order to obtain a wide shunt resistance range for our tests. The range was extended by selecting the photodiodes for high shunt resistance. (The larger the resistivity of the photodiode material, the higher the shunt resistance.) The measured shunt resistances ranged from 0.5 GΩ to 7 GΩ. Only photodiodes with small junction capacitance were selected to obtain small voltage gain (low noise boosting) in ac signal measurements. *C*_J_ is proportional to the photodiode area and can be inversely proportional to either the square or the cube root of the width of the depletion layer [14]. The depletion layer width is proportional to the resistivity of the material. This is why *C*_J_ was measured to be lower on the selected high shunt resistance photodiodes. During the capacitance measurements on the photodiodes the dc voltage drop was less than 13 mV and the ac voltage drop was less than 24 mV. The capacitances were measured in the dark and the diodes were reverse biased. The measured capacitances ranged from 0.18 nF to 0.62 nF. Table 2 also shows the dc voltage gain and *f*_2_ for the 10^10^ V/A signal-gain. They were calculated from the measured *R*_S_ and *C*_J_ values.

The 1/*f* voltage and current noise of the three different types of operational amplifiers are shown in Table 3 for the frequency range between 0.1 Hz and 10 Hz. The manufacturer’s data were helpful only to select candidate operational amplifiers. As noise figures for the small frequency interval (electrical bandwidth) at 9 Hz were not available, it was necessary to test the noise floor experimentally. Both the OPA128LM and the OPA627BM operational amplifiers were tested with all four photodiodes. The OPA111BM was tested only with two different shunt resistances using the S5226-8BQ and the S1227-66BQ photodiodes.

**Fig. 6:**
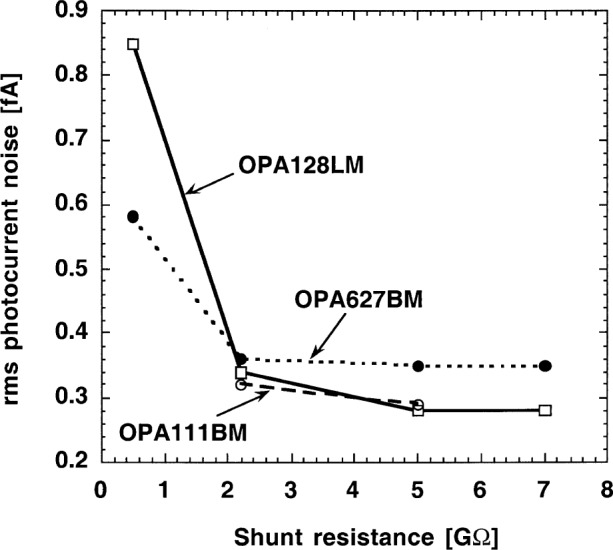
Noise-equivalent photocurrent of photodiode-operational amplifier combinations at a signal-gain of 10^10^ V/A and a chopping frequency of 9 Hz.

Figure 6 shows the measured noise floors of the different photodiode-operational amplifier combinations. These noise floor measurements were made at a current-to-voltage gain of 10^10^ V/A and 9 Hz. The time constant selected on the lock-in amplifier was 3.33 s. The low-pass filters of the lock-in amplifier had a roll-off slope of 20 dB per decade.

For the OPA128LM operational amplifier the output noise decreased to an equivalent rms photocurrent of 0.28 fA for photodiode shunt resistances of 5 GΩ and 7 GΩ. At these high shunt resistances the voltage gain was small enough to make the amplified OA input noise negligible relative to the dominant Johnson noise. The 0.5 GΩ shunt resistance increased the voltage gain by a factor of seven, resulting in a dominating output noise from the input voltage noise. Similar results were obtained with the OPA111BM operational amplifier.

With the OPA627BM operational amplifier a 0.36 fA noise floor was measured for shunt resistances of 2.2 GΩ or larger. This noise floor was 0.08 fA higher than the noise floor with the other two operational amplifiers. As compared to the others, this amplifier had a much larger 1/*f* input current noise and it dominated the OA input noise. With increasing shunt resistance the input voltage noise drop (caused by the input-current noise) increased and the voltage gain for it decreased. The result was a constant 1/*f* noise contribution to the dominating Johnson noise.

**Fig. 7:**
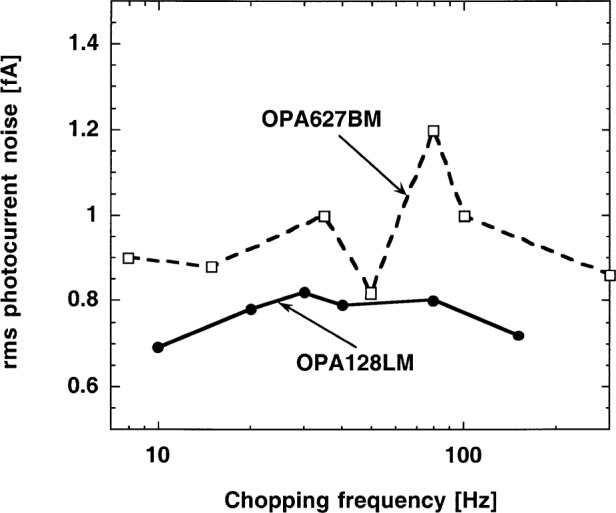
Noise-equivalent photocurrent versus frequency of the silicon radiometer at a signal gain of 10^9^ V/A and a time constant of 3.33 s. The detector was a S5226-8BQ photodiode (*R*_S_ = 2.2 GΩ; *C*_J_ = 410 pF).

The output (total) noise spectrum of the radiometer was measured at different chopping frequencies between 8 Hz and about 200 Hz. The signal gain of the radiometer was lowered to 10^9^ V/A to increase the signal upper roll-off point from 13 Hz to 80 Hz. The time constant of the lock-in amplifier remained 3.33 s. The 2.2 GΩ shunt resistance of the S5226-8BQ photodiode was used in this test. The voltage gain, according to Fig. 4, increases almost two decades from 0.8 Hz to 80 Hz. The upper half of this frequency interval could be tested. As is shown in Fig. 7, the dominating noise (at the radiometer output) is the white Johnson noise, which makes the noise spectrum flat. The measured noise spectrum with the OPA128LM was smoother than with the OPA627BM because of the smaller OA input noise. The input noise of the OPA627BM was dominated by the above-discussed 1/*f* input current noise. In spite of this 1/*f* noise contribution, the noise spectrum remained flat. The smaller voltage gain compensated for the higher 1/*f* noise at the lower frequencies. Because of the flat noise floor at this signal gain, any signal (chopping) frequency can be selected within the tested frequency interval. However, the 3 dB point at 80 Hz will restrict the upper signal frequency limit. If the external feedback capacitor is not connected, this limit can be extended to about 130 Hz. In this case the loop gain at this signal frequency can be too low, resulting in poor current-to-voltage conversion accuracy. To avoid this problem, either a high open-loop gain operational amplifier (e.g., OPA111BM) has to be selected or the partial frequency compensation (e.g., for 80 Hz) has to be applied.

### 5.2 Dark Total Noise Versus Signal Gain

**Fig. 8:**
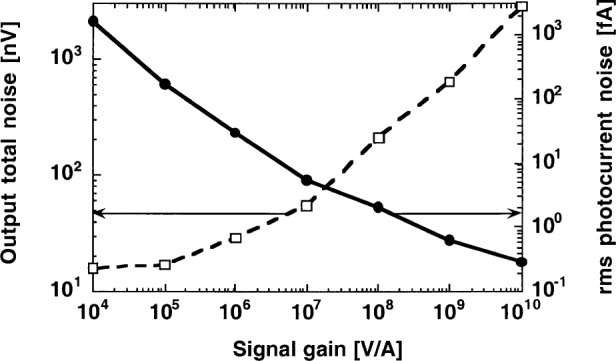
Measured dark noise versus signal gain at 9 Hz and 3.3 s integration time constant. The S1227-66BQ photodiode and the OPA111BM operational amplifier were used in the noise-optimized radiometer.

The optimized radiometer, with the S1227-66BQ (*R*_S_ = 5 GΩ) photodiode and the OPA111BM operational amplifier, was tested for output total noise versus signal-gain performance. Again, the signal (chopping) measurement frequency was 9 Hz and the time constant of the lock-in amplifier was 3.33 s (0.05 Hz bandwidth). The measurement results are shown in Fig. 8. The output total noise is shown by the dashed curve and the noise-equivalent photocurrent is represented by the solid curve. The shape of the total noise curve shows that with high feedback resistances the Johnson noise dominates the output noise. With small feedback resistances, where the Johnson noise is low, the input voltage noise of the amplifier (amplified by the unity voltage gain here) dominates the output noise of the radiometer. The measured 16 nV input voltage noise is a 1/*f* noise which cannot be reduced much by longer integration.

### 5.3 Dark Total Noise Versus Electrical Bandwidth

**Fig. 9:**
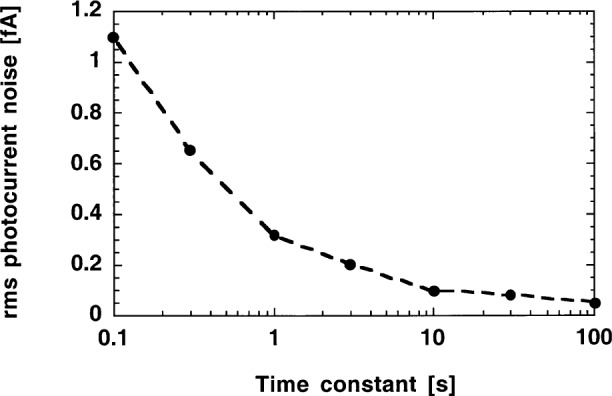
Noise-equivalent photocurrent versus time constant of the digital lock-in amplifier for the S1226-8BQ photodiode and the OPA128LM operational amplifier at 9 Hz chopping frequency and 10^10^ V/A signal gain.

The Johnson noise in Fig. 8 can be further reduced by longer integration. The noise-optimized radiometer with the S1226-8BQ (*R*_S_ = 7 GΩ) photodiode and the OPA128LM operational amplifier was tested at a signal gain of 10^10^ V/A and a measurement frequency of 9 Hz. A digital lock-in amplifier (Stanford SR830) was used in this test. The time constant of the lock-in amplifier was changed between 100 s and 0.1 s. The waiting time was three times the time constant before each dark noise measurement. The measurement results are shown in Fig. 9.

The dark noise floor was 0.1 fA with the 10 s time constant. This noise is equal to the earlier reported dc dark noise obtained with a measurement time of 400 s [4]. The present 9 Hz (ac) noise test needed a 40 s total measurement time to obtain the same noise-equivalent photocurrent. At the 100 s time constant the noise equivalent photocurrent decreased to 0.05 fA. The noise equivalent power (NEP), which is the ratio of the 0.05 fA to the 0.36 A/W peak responsivity of the diode, is equal to 0.14 fW. This test required a total measurement time of 400 s. The roll-off slopes of the low-pass filters, within the digital lock-in amplifier, were always selected to 80 dB per decade (24 dB per octave). This very steep roll-off slope produced a well-defined electrical bandwidth.

The Johnson noise could be calculated from *R*_S_ = 7 GΩ and *R* = 10 GΩ. The parallel connection of *R* and *R*_S_ gave a source resistance of *R*_SO_ = 4.1 × 10^9^ Ω. The rms noise voltage of the source resistance, at the output of the radiometer, was

$$V_{N}={\left(4kTR_{\text{SO}}\Delta f\right)}^{1/2}=1.81\;\text{μV},$$ (8)

where *k* = 1.38 × 10^−23^ J/K is the Boltzmann constant, *T* = 300 K was the temperature of the radiometer during the test, and Δ*f* = 0.048 Hz was the measurement bandwidth as calculated from the 3.33 s time constant of the lock-in amplifier used in the noise measurements of Fig. 6.

The 0.28 fA noise-equivalent photocurrent in Fig. 6 was calculated from the measured 2.8 μV radiometer output noise voltage and the 10^10^ V/A photocurrent-to-voltage gain. Δ*f* in that measurement was larger than in Eq. (8) because of the shallow (20 dB per decade) slope of the low-pass filter. That measurement was repeated with the digital lock-in amplifier, using a 3 s time constant with a roll-off slope of 80 dB per decade. With this time constant, the Δ*f* = 0.053 Hz bandwidth resulted in a calculated Johnson noise voltage of 1.9 μV. In accordance with Fig. 9, the measured value was 2 μV. This noise voltage corresponds to a 0.2 fA photocurrent noise at a signal gain of 10^10^ V/A. The 80 dB per decade roll-off still does not give a perfect square shape for the measurement bandwidth. The 10 % (coverage factor *k* = 2) uncertainty of these Johnson noise determinations is well within the 38 % relative expanded uncertainty (*k* = 2) of the ac noise measurements.

## 6. DC Dark Noise Measurements

**Fig. 10:**
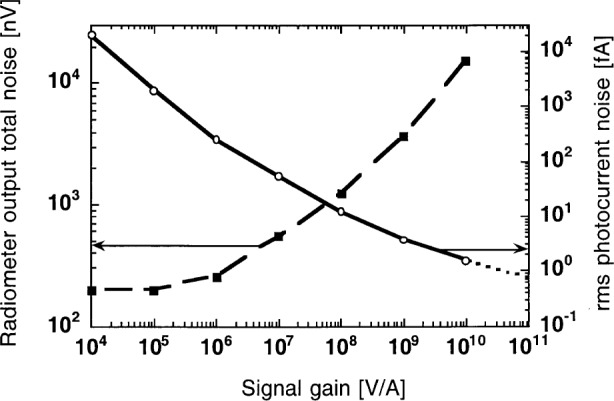
The dc dark noise of the radiometer noise-optimized in the ac mode (with S1226-8BQ photodiode and OPA128LM operational amplifier) versus signal gain. The integration time is 1.7 s.

The silicon radiometer, noise-optimized with the S1226-8BQ photodiode (*R*_S_ = 7 GΩ) and the OPA128LM operational amplifier in the ac mode, was tested in the dc measurement mode as well. As in our earlier reported dc measurements [4], the bandwidth was again 0.3 Hz determined by the 100 power-line cycle integration time (1.7 s) of the dc digital voltmeter connected to the output of the radiometer. The temperature of the radiometer during the dc test was controlled to 25 °C ± 0.02 °C. The increase in the standard deviation of the measured noise data points due to drift was negligible. The dc dark noise measurement results are shown in Fig. 10.

At the two lowest signal gains (10^4^ V/A and 10^5^ V/A) the output total noise of the radiometer was dominated by the 0.2 μV input voltage noise of the OA. This 1/*f* noise fits to the earlier reported 0.2 μV to 1 μV noise interval [4] measured on several OPA128LM operational amplifiers. It should be noted that the ac and the dc noise versus signal-gain tests were made with different photodiode-amplifier pairs. However, before the tests (both ac and dc), the two radiometer combinations were optimized to the same noise floor (0.28 fA) at 9 Hz, as discussed in Sec. 5.1.

At the highest (10^10^ V/A) signal gain of the radiometer, the noise-equivalent photocurrent was 1.5 fA. The noise-equivalent photocurrent increased to about 20 pA at the lowest (10^4^ V/A) signal gain. When the equivalent photocurrent noise curve was extrapolated to the 10^11^ V/A gain, a noise floor of about 0.7 fA was obtained, equal to the noise floor of our previous dc measurements (using the same signal gain and the same 0.3 Hz bandwidth) [4]. The lower noise floor at 10^11^ V/A can be utilized only at very slow measurements because the settling time constant at this gain is 16.2 s [9]. At 10^10^ V/A signal gain the settling time constant is shorter than one power line cycle (16.7 ms). This three orders of magnitude settling-time difference rejects the application of the 10^11^ V/A signal gain from fast measurements. The expanded relative uncertainty (coverage factor *k* = 2) of our dc noise measurements was 25 %.

## 7. AC and DC Dark Noise Comparison

In dc measurements, the noise-boosting effect (shown in Fig. 4) can be avoided if we restrict the measurement frequency and bandwidth to a limit which is lower than the frequency where the voltage-gain increase begins. We used this method in our previous work [4] where a 400 s measurement time (1.25 mHz bandwidth) eliminated the noise-boosting effect at the 10^11^ V/A signal gain and equalized the resistor noise to the 1/*f* noise.

Another method to eliminate (or decrease) noise boosting is to apply frequency compensation for the different signal gains of the radiometer. In a typical example, where the shunt resistance is 6.5 GΩ and the junction capacitance is 1.3 nF, full-frequency compensation will decrease the signal 3 dB roll-off points to 19 mHz at the two highest signal gains. (This requires a 85 pF capacitor parallel to *R* = 10^11^ Ω and 850 pF parallel to *R* = 10^10^ Ω.) Again, the result would be very slow signal measurements at high signal gains. However, full-frequency compensation is not needed at high signal gains because the large feedback resistance and shunt resistance make the Johnson noise dominate (as discussed in Sec. 5.1) relative to the amplified (boosted) input noise.

In both dc and ac measurements, partial frequency compensation give the best results. The voltage gains are decreased (with the partial compensation) by lowering *f*_1_ (closer to *f*_2_) until the signal response is not too low for the signal frequency. This was the design consideration for our test radiometer (as described in Sec. 3) where the signal roll-off frequency was at least 10 times the chopping frequency. As is shown in Figs. 3 and 4, the value of the compensating *C*_f_ capacitor depends on the chosen signal gain (*R*).

The input voltage noise at 9 Hz was 16 nV with the OPA111BM (as shown in Fig. 8) and 23 nV with the OPA128LM. The 1/*f* noise of each amplifier at this measurement frequency is much smaller than at very low (dc) frequencies. Earlier [4], a 50 nV to 200 nV 1/*f* input noise interval was reported on several OPA111BM operational amplifiers at very low frequencies. The 1/*f* noise interval was higher (about 0.3 μV to 1 μV) for the OPA128LM.

The decrease of the 1/*f* noise at higher frequencies made it possible to increase the signal measurement frequency from dc to 9 Hz. At 9 Hz the noise-boosting effect was still significant. However, the feedback resistor (*R*) could be lowered by a factor of 10, relative to the previously reported *R* = 100 GΩ in the most sensitive dc measurements [4]. The result of the smaller *R* was decreased voltage gain (*A*_V_) and resistor noise.

Figure 8 shows that the dark noise floor measured at 9 Hz is about a decade lower than the dc dark noise floor shown in Fig. 10. The silicon radiometers in the ac and dc noise floor measurements were noise optimized (in ac mode) to the same 0.28 fA rms current level (as described in Sec. 5.1). The measurement times are reasonably short (a few seconds) in both cases. At 9 Hz, the integration time to obtain the same 10^−16^ A noise floor (as in the previous dc measurements) became shorter because of the lower resistor noise. Using the radiometer with the OPA128LM operational amplifier and a 30 s integration time constant (as shown in Fig. 9), an output rms 1/*f* noise of (0.93^2^ − 0.6^2^)^1/2^ μV = 0.71 μV could be calculated. The 0.6 μV was the calculated rms Johnson noise and the 0.93 μV was the total rms output noise measured by the lock-in *X* channel (as shown in Table 1). The *X*-channel noise was used because it was free of the residual output offset in *M*. The calculated voltage gain (at 9 Hz) was 710 nV / 23 nV = 31. The relative uncertainty (coverage factor *k* = 2) of this voltage gain determination was 66 %. In this ac measurement, the Johnson noise was roughly equalized to the amplified 1/*f* noise. The noise equivalent photocurrent (from the lock-in magnitude *M*) was 8 × 10^−17^ A. According to Fig. 9, further increase of the lock-in integration time constant will not yield a significant decrease in the output total noise of the radiometer. The lowest noise-equivalent photocurrent of 5 × 10^−17^ A was measured with an integration time constant of 100 s. The total duration of this ac measurement was 400 s.

## 8. Conclusion

An experimentally verified noise analysis for silicon photodiode current meters of improved dynamic characteristics has been described. Transimpedance gain, loop gain, and voltage gain were optimized versus frequency using partial frequency compensation for the different signal gains. The effect of the Johnson noise, the OA input voltage noise, and the current noise of both the OA input current and the photocurrent were discussed. The transimpedance (current-to-voltage) gains of the test radiometer were measured versus frequency. The noise floor was optimized at a signal chopping frequency of 9 Hz by testing the combinations of four photodiodes of different shunt impedance and three selected operational amplifiers. The dominating Johnson noise at the tested 10^10^ V/A signal gain was further decreased by increasing the integration time constant of the ac signal measurements. At an integration time constant of 30 s (after 90 s wait), the Johnson noise was equalized to the amplified 1/*f* noise of the operational amplifier, resulting in an rms noise equivalent photocurrent of 0.08 fA. This photocurrent noise corresponds to an NEP of 2.2 fW at the 0.36 A/W peak responsivity of the photodiode. Earlier [4], a 400 s measurement (averaging) time was needed in the dc measurement mode to obtain an rms noise floor of 0.1 fA.

The silicon radiometer optimized in the ac measurement mode was tested in the dc measurement mode as well. The dc tests verified that a radiometer optimized for ac measurements may also have an optimum performance in the dc mode. Integrating-type digital voltmeters measured the radiometer output voltages for the duration of 100 power line cycles (1.7 s) to avoid long measurement (averaging) times. Also, the signal gain of the radiometer was maximized to 10^10^ V/A to avoid long settling times. The dc test resulted in a practical rms photocurrent sensitivity limit of 1.5 fA. In contrast, the practical sensitivity limit in the ac measurement mode (with a 10 s integration time constant) was 0.1 fA. The conclusion from comparing the ac and the dc photocurrent measurement modes is that well-designed ac radiometers can measure weaker optical signals with shorter measurement times.

The described frequency- and noise-optimized silicon photodiode current meters can be used as building blocks of high-sensitivity radiometers, photometers, pyrometers, and colorimeters. These radiometers have a signal (radiant power or photocurrent) dynamic range of greater than 14 decades. The discussed ac (including dc) noise-optimization method can be extended for ultraviolet and near-infrared photodiode current meters as well.

## Figures and Tables

**Table 1:** Lock-in *X* and *Y* output noise voltages and magnitudes *M*, for the dark S1226-8BQ photodiode and the OPA128LM operational amplifier at 9 Hz chopping frequency and 10^10^ V/A signal gain

Lock-in time constant (s)	*M* (μV)	*X* (μV)	*Y* (μV)
1	3.2	3.2	3.5
30	0.78	0.93	0.81

**Table 2:** The tested silicon photodiodes and the calculated voltage gain parameters

Model	*R*_S_(GΩ)			*C*_J_(nF)		$A_{V_{0}}$	*f*_2_(Hz)

	Data Book		Measured in the dark	Data Book Typical	Measured in the dark	For *R* = 10^10^ Ω and measured *R*_S_ and *C*_J_	
	Min.	Typical					
S1336-8BQ	0.1	0.4	0.5	0.38	0.18	21	1.9
S5226-8BQ	0.1	1	2.2	0.43	0.32	5.6	0.3
S1227-66BQ	0.2	1	5	0.95	0.62	3	0.08
S1226-8BQ	0.2	1	7	0.95	0.56	2.4	0.07

**Table 3:** Catalog data of OA 1/*f* noises from 0.1 Hz to 10 Hz (all voltages and currents are peak-to-peak)

OPA128LM		OPA627BM		OPA111BM	
Voltage	Current	Voltage	Current	Voltage	Current
4 μV	2.3 fA	1.6 μV (0.6 typical)	60 fA (30 typical)	2.5 μV	12 fA (7.5 typical)

## Footnotes

1 Certain commercial equipment, instruments, or materials are identified in this paper to foster understanding. Such identification does not imply recommendation or endorsement by the National Institute of Standards and Technology, nor does it imply that the materials or equipment identified are necessarily the best available for the purpose.
